# Development and application of neonatal physiology‐based pharmacokinetic models of amikacin and fosfomycin to assess pharmacodynamic target attainment

**DOI:** 10.1002/psp4.13097

**Published:** 2024-01-03

**Authors:** Christopher A. Darlow, Neil Parrott, Richard W. Peck, William Hope

**Affiliations:** ^1^ Antimicrobial Pharmacodynamics and Therapeutics, Department of Pharmacology University of Liverpool Liverpool UK; ^2^ Pharmaceutical Sciences, Roche Pharma Research and Early Development, Roche Innovation Centre Basel Basel Switzerland

## Abstract

Antimicrobial resistance increasingly complicates neonatal sepsis in a global context. Fosfomycin and amikacin are two agents being tested in an ongoing multicenter neonatal sepsis trial. Although neonatal pharmacokinetics (PKs) have been described for these drugs, the physiological variability within neonatal populations makes population PKs in this group uncertain. Physiologically‐based pharmacokinetic (PBPK) models were developed in Simcyp for fosfomycin and amikacin sequentially for adult, pediatric, and neonatal populations, with visual and quantitative validation compared to observed data at each stage. Simulations were performed using the final validated neonatal models to determine drug exposures for each drug across a demographic range, with probability of target attainment (PTA) assessments. Successfully validated neonatal PBPK models were developed for both fosfomycin and amikacin. PTA analysis demonstrated high probability of target attainment for amikacin 15 mg/kg i.v. q24h and fosfomycin 100 mg/kg (in neonates aged 0–7 days) or 150 mg/kg (in neonates aged 7–28 days) i.v. q12h for Enterobacterales with fosfomycin and amikacin minimum inhibitory concentrations at the adult breakpoints. Repeat analysis in premature populations demonstrated the same result. PTA analysis for a proposed combination fosfomycin‐amikacin target was also performed. The simulated regimens, tested in a neonatal sepsis trial, are likely to be adequate for neonates across different postnatal ages and gestational age. This work demonstrates a template for determining target attainment for antimicrobials (alone or in combination) in special populations without sufficient available PK data to otherwise assess with traditional pharmacometric methods.

**Study Highlights**

**WHAT IS THE CURRENT KNOWLEDGE ON THE TOPIC?**

Physiologically‐based pharmacokinetic (PBPK) models had previously been developed to predict neonatal drug exposures, but not with these drugs. Drug exposures were previously determined in this group only through population PK (PopPK) models.

**WHAT QUESTION DID THIS STUDY ADDRESS?**

This study demonstrates development of validated neonatal PBPK models for amikacin and fosfomycin and predicts the neonatal drug exposures of fosfomycin and amikacin in neonates of different postnatal and gestational age to determine probability of target attainment in these groups.

**WHAT DOES THIS STUDY ADD TO OUR KNOWLEDGE?**

This study gives greater confidence in the selected neonatal regimens of fosfomycin and amikacin, currently being tested in a multicenter neonatal sepsis trial.

**HOW MIGHT THIS CHANGE DRUG DISCOVERY, DEVELOPMENT, AND/OR THERAPEUTICS?**

The approach demonstrated here provides a template for predicting antimicrobial probability of target attainment in special populations (such as neonates) that do not have PK data or PopPK models to determine this using traditional methods.

## INTRODUCTION

Neonatal sepsis is a serious infection of newborns^1^ and is associated with an estimated 680,000 deaths annually.^2^ The World Health Organization currently recommends a narrow spectrum β‐lactam in combination with gentamicin for the treatment of neonatal sepsis.^3^ However, there is increasing evidence of widespread antimicrobial resistance to this regimen in multicenter epidemiology studies.^4^ As a result of this, the Global Antibiotic Research and Development Partnership has been leading development of alternate antimicrobial regimens that can be deployed in a range of healthcare settings.^5^

Fosfomycin and amikacin are potential candidates for treatment of neonatal sepsis in highly prevalent resistance settings.^6^ Both are being tested in combination in an ongoing neonatal sepsis trial (NeoSep1 – ISRCTN number ISRCTN48721236). Although the pharmacokinetics (PKs) of both have been described in neonates,^7^, ^8^ the physiological variability within the neonatal population,^9^ particularly in prematurity, mean that the drug exposures of these drugs have only been determined within the limited demographic frame of the populations in the relevant PK studies.

Physiologically‐based PK (PBPK) modeling has the capability to simulate drug PKs in specific populations, including pediatric and neonatal populations, and to recover the physiological variability within these special populations. The mechanistic nature of PBPK modeling means that drug exposures can be predicted in physiological contexts not captured by datasets from traditional PK trials or population PK (PopPK) models derived from them.

Here, we develop neonatal PBPK models for both amikacin and fosfomycin and predict the drug exposures for fosfomycin and amikacin across a range of neonatal demographics.

## METHODS

### Pharmacokinetic data collection

Papers containing PK data for both agents were identified through a search of Medline using the terms “amikacin pharmacokinetics” and “fosfomycin pharmacokinetics” up until December 2021. All papers were screened for relevance, with studies detailing time‐concentration data for either amikacin or fosfomycin for any age group identified. PK data were extracted along with any relevant demographic data. Where individual numerical values were not provided, data were extracted from the figures detailing time‐concentration profiles using WebPlotDigitizer (https://automeris.io/WebPlotDigitizer/).

### PBPK model development

All PBPK work was performed using the version 22 Simcyp Simulator (Certara), a commercial software platform for population PBPK.

For each drug, an initial PBPK model in the adult population was developed by integrating available data on the known characteristics of each drug including physical–chemical properties, elimination routes, and enzyme/transporter activities. Performance of the model was checked visually against a typical adult PK dataset and if a mismatch was observed, the model was revised via justified optimization of a limited number of parameters within their ranges of uncertainty. Systematic visual and quantitative assessment was performed by comparing the output for 100 simulated individuals to each available adult PK dataset where the drug regimen, demographics, and physiological state of the participants of each study could be readily replicated by the simulator.

Specific quantitative measures included observed: predicted ratios of maximum plasma concentration (*C*_max_) and area under the curve (AUC). For *C*_max_, the PBPK model output at the time of the first measured concentration was used to avoid comparisons of the post‐bolus high concentrations simulated by PBPK models, but not captured in typical PK studies. Simulated AUC values were calculated using Simcyp whereas for the observed data they were calculated using the linear trapezoid method in GraphPad Prism (GraphPad Software).

Each measure was summarized as the geometric mean across studies weighted by the number of participants in each study. This was calculated using the equation:

$$w{\bar{x}}_{\mathrm{Geo}}=\mathrm{Exp}\left(\left(\frac{1}{\sum n}\right)*\sum \left(n*\ln\left(\frac{x}{y}\right)\right)\right)$$

where $w{\bar{x}}_{\mathrm{Geo}}$ = weighted geometric mean; *n* = number of individuals in each PK trial; *x* = observed PK parameter; and *y* = predicted PK parameter. A model was deemed successfully validated if this measure was within a 0.25‐fold error range of 1 (i.e., weighted geometric mean ratios of 0.8–1.25).

Once successfully validated, the adult model was scaled to predict the pediatric PK using the known age‐dependency of physiology and ontogenetic relationships within Simcyp. For pediatrics, the same visual and quantitative model validation steps were performed against available PK datasets for children as have been described for the adult model. After successful model refinement and validation in the older children, the pediatric model was used to simulate for neonates, again applying the Simcyp default age‐related scaling and ontogeny functions. Further refinement and validation against neonatal PK datasets were performed and then the final validated neonatal model was used for simulations of target attainment.

### PBPK simulations

Simcyp simulations were performed with the final fosfomycin and amikacin models using simulated regimens and postnatal age (PNA) groups (0–7 days and 7–28 days) selected to replicate the established amikacin dosing in neonates^10^ and the PNA‐specific regimens suggested by Kane et al.^8^ All simulations were performed with term neonates (using the “Pediatric” pre‐set Simcyp population) and neonates with a gestational age of 30 weeks (using the “Pre‐term” pre‐set Simcyp population) to assess the predicted effects of prematurity, up to a pragmatically selected PNA ceiling of 28 days in both populations.

Each simulated cohort consisted of 1000 individual neonates with co‐administered fosfomycin and amikacin over a period of 7 days. Subjects were redefined every 6 h to capture the physiological changes of neonates over the simulation period. Population output for the simulated AUC and *C*_max_ of each drug was then used to determine the probability of target attainment applying the PK/pharmacodynamic (PD) targets for fosfomycin, amikacin, and the combination of both.^6^, ^11^, ^12^, ^13^ Specifically, these PK/PD targets are: (1) the fosfomycin AUC:minimum inhibitory concentration (MIC) targets of greater than 21.4 and greater than 62.5 for stasis and 1−log_10_ CFU/mL reduction, respectively^11^; (2) the classically used amikacin *C*_max_:MIC target of greater than 10 for clinical success^12^; (3) _T_he newly determined amikacin AUC:MIC targets of greater than 23 and greater than 82 for stasis and 1−log_10_ CFU/mL reduction, respectively^13^; and (4) a proposed fosfomycin‐amikacin combination target of MIC_Fosfomycin_*MIC_Amikacin_ greater than 2709.5 for a 5‐log_10_ CFU/mL reduction.^6^

## RESULTS

### Fosfomycin PBPK model development

**FIGURE 1:**
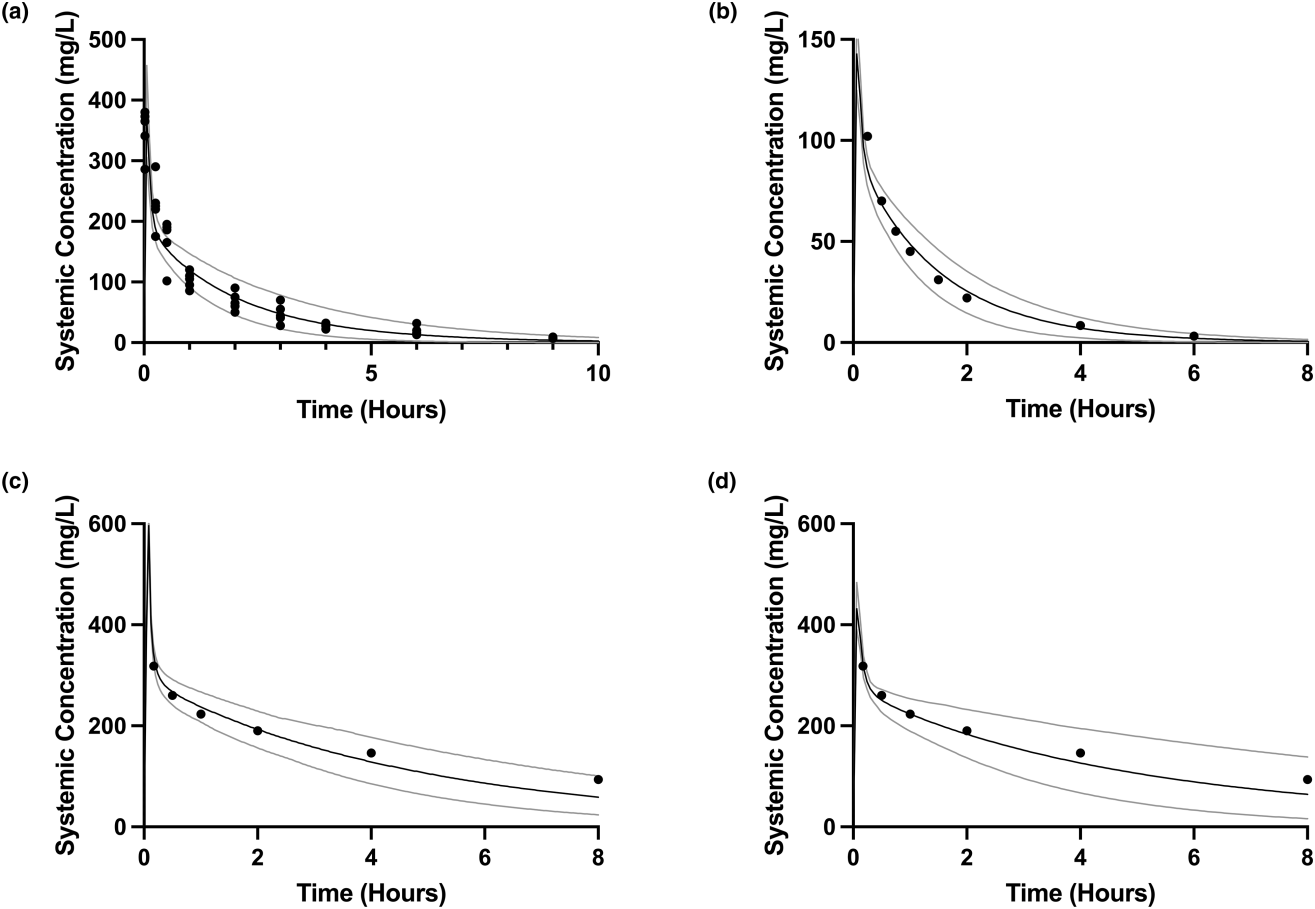
Visual validation of fosfomycin adult, pediatric, and neonatal PBPK models. The black solid line indicates the mean systemic concentration predicted by the PBPK model, with gray solid lines indicating 5th and 95th centiles, from 100 simulated individuals for each validation. The overlying symbols indicate observed concentrations from the test dataset. (a) Adult fosfomycin PBPK model simulating a 50 mg/kg i.v. bolus in adult healthy volunteers, with overlaid individual observed data from Segre et al.^17^ (b) Pediatric fosfomycin PBPK model simulating a 25 mg/kg i.v. bolus in children aged 3–8 years, with overlaid population mean observed data from Guggenbichler et al.^27^ (c) Initial neonatal fosfomycin PBPK model simulating a 100 mg/kg i.v. bolus in neonates aged 0–23 days, with overlaid population mean observed data from Kane et al.^8^ (d) Adjusted neonatal fosfomycin PBPK model (with Kp scalar of 1.2) simulating 100 mg/kg i.v. bolus in neonates aged 0–23 days, with overlaid population mean observed data from Kane et al.^8^ PBPK, physiologically‐based pharmacokinetic.

Using the physical–chemical properties and pharmacological parameters for fosfomycin, which is an ampholyte (Table 1), the best performing tissue distribution prediction was a full PBPK model using the Gaohua model (Model 3 in Simcyp),^14^ which predicted a steady‐state volume of distribution (*V*_ss_) of 0.24 L/kg. Clearance in the model was only via renal elimination with no hepatic contribution. The renal value was set to the reported renal clearance by Dijkmans^15^ which was identical to the normal glomerular filtration rate. Simulated PK closely matched the test dataset (Figure 1a) and validated well against the available adult fosfomycin PK datasets^16^, ^17^, ^18^, ^19^, ^20^, ^21^, ^22^, ^23^ (Table S1), with weighted geometric mean AUC and *C*_max_ ratios of 0.96 and 0.97, respectively.

**TABLE 1:** Physical–chemical properties and relevant pharmacokinetics parameters for a healthy 30 year old adult for fosfomycin and amikacin.

Parameter	Fosfomycin value	Amikacin value
Molecular weight (g/mol)	138	586.6
pKa 1/2	1.25/4.3 (ampholyte)^24^	6.7/8.4 (diprotic base)^25^
Lipophilicity (LogP)^a^	−1.6	−8.6
Blood/plasma ratio^b^	0.68	0.55
Fraction unbound	0.99^16^	0.964^26^
Hepatic clearance (L/h)	0^19^	0^26^
Renal clearance (L/h)	7.68^19^	6.93^26^

^a^ Parameter value predicted from PubChem.

^b^ Denotes parameters predicted using other physical–chemical properties with the Simcyp simulator.

As the performance of the adult fosfomycin PBPK model was within acceptable limits, we scaled the model without structural modification using Simcyp defaults for physiological and ontogenetic changes, to create a pediatric PBPK model. Simulations with this model visually matched the test dataset well (Figure 1b) and validated well against the available pediatric fosfomycin PK datasets^27^, ^28^ (Table S2) with weighted geometric mean AUC and *C*_max_ ratios of 0.98 and 1.00, respectively.

We further scaled this acceptably performing pediatric PBPK model without structural modification to the neonatal population. Simulations with this final neonatal model visually matched the test dataset well (Figure 1c). However, validation of the model against fosfomycin neonatal PK datasets^8^, ^27^, ^29^, ^30^ gave weighted geometric mean AUC and *C*_max_ ratios of 0.96 and 0.71 respectively, with a systematic overestimation of *C*_max_ (Table S3). To correct this apparent failure of prediction distribution, the model was refined to improve the *C*_max_ prediction visually against the Kane et al. dataset,^8^ with an introduced partition scaling factor (Kp) of 1.2. A repeat validation gave weighted mean AUC and *C*_max_ ratios of 1.03 and 0.87 (Table S4). Although there were some individual outliers, the overall performance of this model was considered to be acceptable and was used for further simulations.

### Amikacin PBPK model development

Using the physical–chemical properties and pharmacological parameters for amikacin, which is a hydrophilic strong base (Table 1), the best performing tissue distribution model was a full PBPK model using the Gaohua prediction method (Model 3 in Simcyp).^14^ However, *V*_ss_ was still systematically overestimated at 1.04 L/kg compared to the observed value ranges of 0.17–0.29 L/kg^26^, ^31^, ^32^ and a scaling factor (Kp scalar) of 0.17 was therefore applied to the tissue partitioning. Clearance was modeled as renal elimination only with no hepatic contribution based on the reported data of Clarke et al.^26^

**FIGURE 2:**
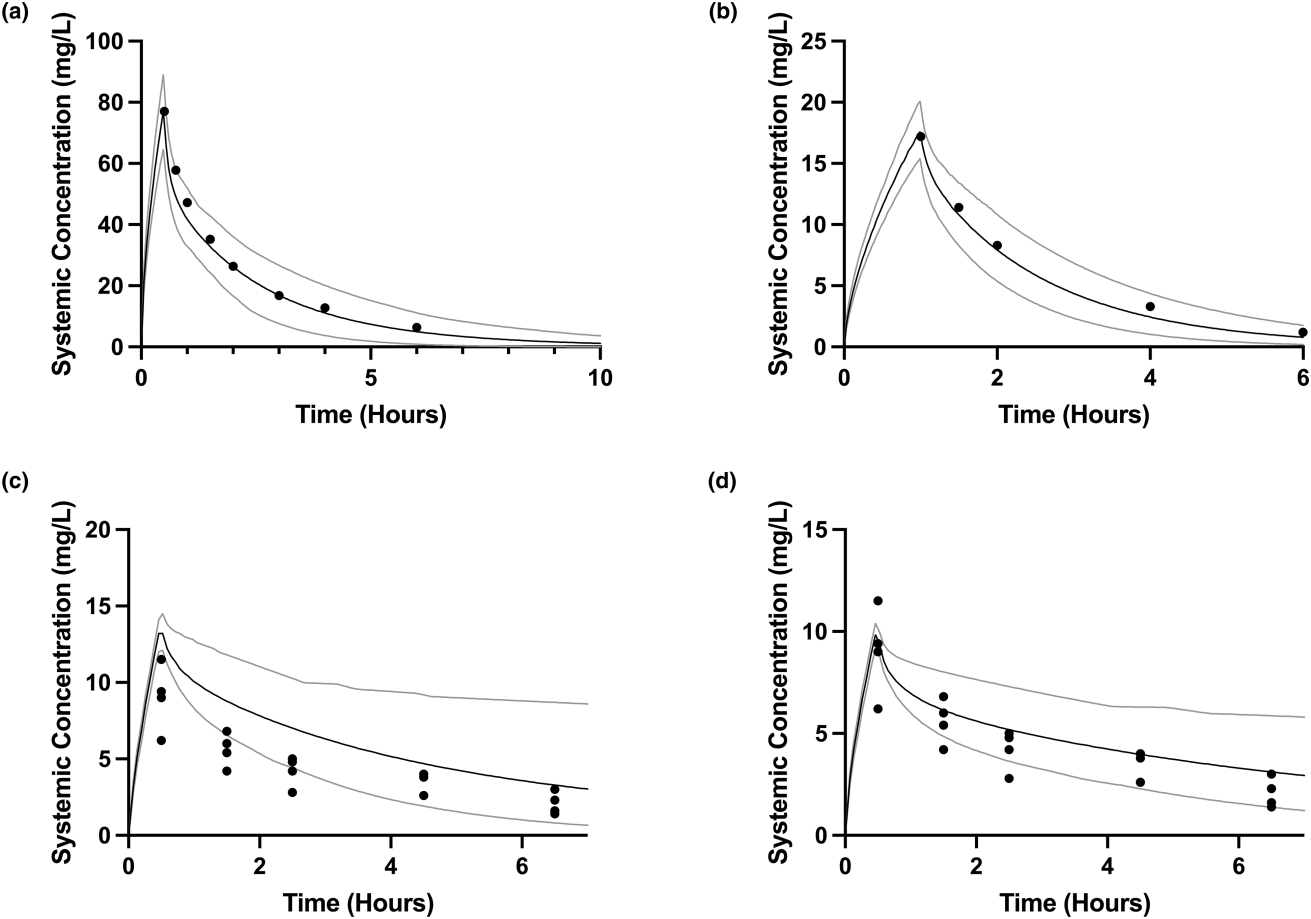
Visual validation of amikacin adult, pediatric, and neonatal PBPK models. The solid line indicates the mean systemic concentration predicted by the PBPK model, with gray solid lines indicating 5th and 95th centiles, from 100 simulated individuals for each validation. The overlying symbols indicate observed concentrations from the test dataset. (a) Adult amikacin PBPK model simulating a 7.5 mg/kg i.v. infusion over 30 min in adult healthy volunteers, with overlaid population mean observed data from Garraffo et al. (b) Pediatric amikacin PBPK model simulating a 5 mg/kg i.v. infusion over 60 min in children aged 1–16 years, with overlaid population mean observed data from Cleary et al.^35^ (c) Neonatal fosfomycin PBPK model simulating a 3 mg/kg i.v. infusion over 30 min in neonates aged 2 – 8 days using a Kp scaler of 0.17, with overlaid individual observed data from Nishimura et al.^36^ (d) As panel (c), but using a PBPK model with a Kp scalar of 0.325. PBPK, physiologically‐based pharmacokinetic.

With the use of this Kp scalar the model performed well against the test dataset (Figure 2a). This model was validated against the available adult amikacin PK datasets with healthy volunteers^26^, ^31^, ^32^, ^33^, ^34^ (Table S5) with weighted geometric mean AUC and *C*_max_ ratios of 1.21 and 1.10, respectively.

As the performance of the adult amikacin PBPK model was within acceptable limits, we scaled the model without structural modification, to create a pediatric PBPK model. Simulations with this model visually matched the test data set well (Figure 2b) and validated against the available pediatric amikacin PK datasets^35^, ^37^, ^38^, ^39^ (Table S6) with weighted geometric mean AUC and *C*_max_ ratios of 1.13 and 1.08, respectively.

We further scaled this pediatric PBPK model to the neonatal population to create a neonatal PBPK model. However, this model performed poorly on visual validation (Figure 2c), and showed gross overestimation of *C*_max_ and AUC_0‐7h_. The observed volume of distribution (*V*_d_) is ~0.4 L/kg^40^ in neonates in contrast to the predicted value of 0.25 L/kg with this model. Therefore, the Kp scalar was adjusted to 0.325 for the neonatal PBPK model to match the empirically observed *V*_d_ with renal clearance left unchanged.

This adjusted neonatal model visually matched the test dataset well (Figure 2d) and validated against the available neonatal amikacin PK datasets^36^, ^38^, ^39^, ^41^, ^42^ (Table S7) with weighted geometric mean AUC and *C*_max_ ratios of 0.98 and 0.93, respectively. Although there were some individual outliers, the overall performance of this model was acceptable, and was used for further simulations.

### Simulation of neonatal drug exposure

**FIGURE 3:**
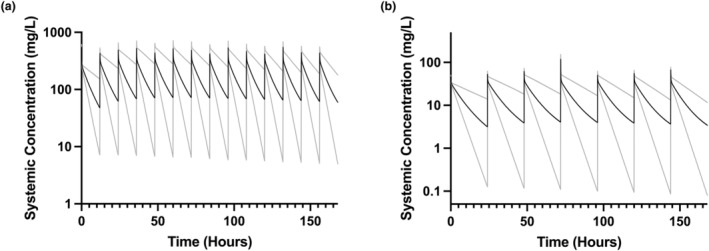
PBPK model output from a simulation of 1000 term neonates aged 0–7 days receiving fosfomycin 100 mg/kg i.v. bolus q12h (a) and amikacin 15 mg/kg i.v. bolus q24h (b), with simulation data sampled every 5 min. The variable *C*_max_ values are due, in part, to the simulated ontogeny and growth of each individual neonate over the simulation time period. *C*_max_, maximum plasma concentration; PBPK, physiologically‐based pharmacokinetic.

Using the developed neonatal PBPK models for fosfomycin and amikacin described above, simulations of drug exposures were conducted with Simcyp using clinically appropriate regimes in different neonatal age groups (Fosfomycin 100 mg/kg i.v. bolus q12h and amikacin 15 mg/kg q24h i.v. bolus)^8^, ^10^, ^43^ in term neonates aged 0–7 days and 7–28 days (*n* = 1000 per cohort). Additionally, as fosfomycin 150 mg/kg q12h i.v. bolus has been recommended for neonates aged greater than 7 days in a recent study,^8^ this regimen in combination with amikacin 15 mg/kg q24h i.v. bolus was simulated too in neonates aged 7–28 days. All simulations were repeated for pre‐term neonates, with a gestation of 30 weeks. Figure 3 shows the simulation output for term neonates aged 0–7 days.

**FIGURE 4:**
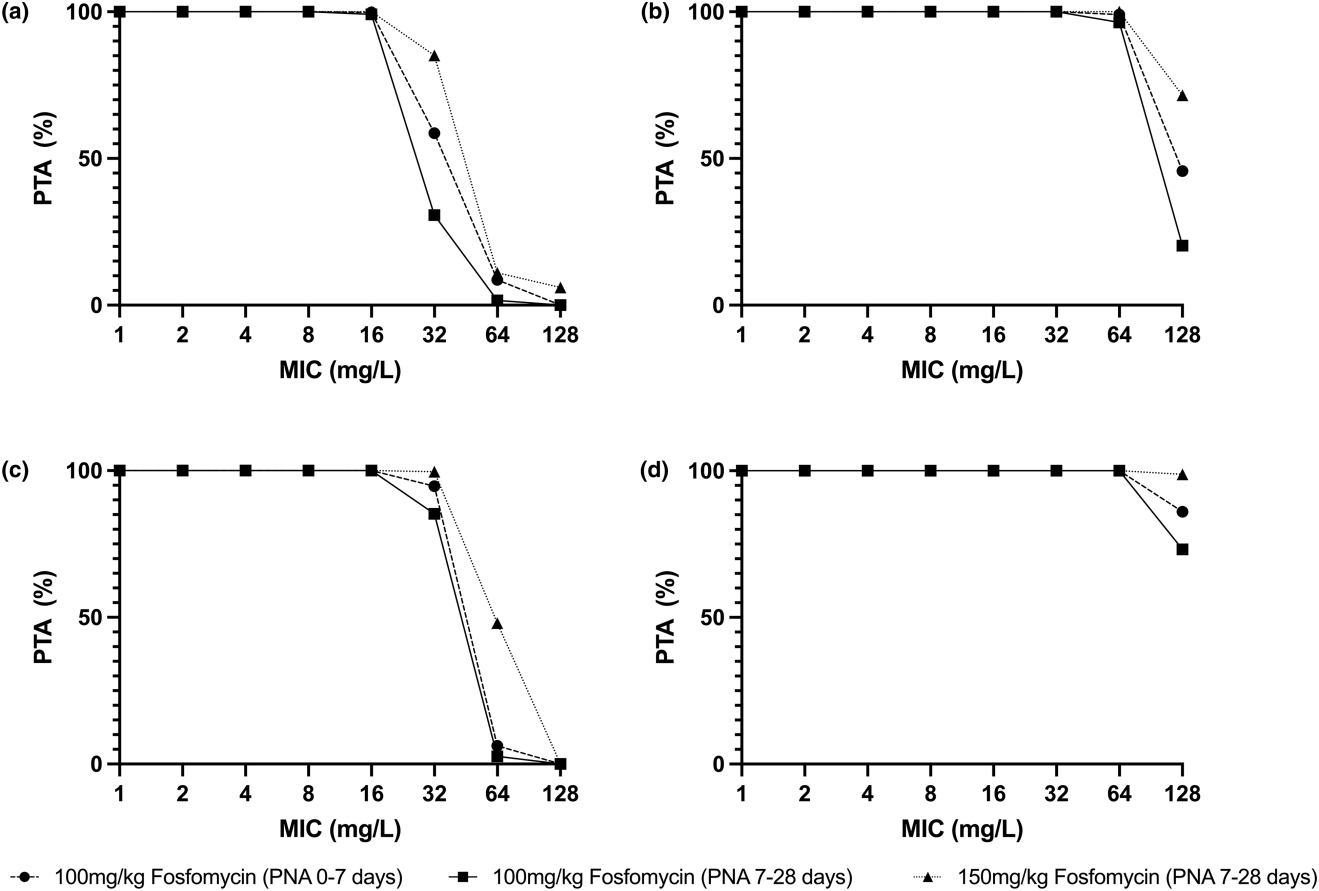
Probability of target attainment for fosfomycin monotherapy for the regimens 100 mg/kg and 150 mg/kg i.v. q12h in neonates of PNA 0–7 days and 7–28 days. Panels (a and c) represent the target attainment for bacterial 1−log_10_ CFU/mL reduction (AUC:MIC >82). Panels (b and d) represent the target attainment for bacterial stasis (AUC:MIC >23). Panels (a and b) are determined in term neonates. Panels (c and d) are determined in pre‐term neonates born at 30 weeks gestation. AUC, area under the curve; MIC, minimum inhibitory concentration; PNA, post‐natal age; PTA, probability of target attainment.

With these exposures, probability of target attainment (PTA) was calculated for monotherapy targets of both drugs^11^, ^12^, ^44^ and a proposed combination target^6^ for an Enterobacterales pathogen with varying MICs to both agents. The fosfomycin monotherapy PTA analysis for both 100 mg/kg and 150 mg/kg q12h in term and premature neonates is shown in Figure 4. Here, the 1−log_10_ CFU reduction target (AUC:MIC >82)^11^ was met for all regimens/PNA combinations at a fosfomycin MIC of 16 mg/L, but variously failed to achieve 95% attainment levels in term infants for MICs above this (Figure 4a). There was greater predicted attainment in premature neonates, with 100 and 150 mg/kg regimens achieving 94.7% and 99.6% attainment for premature infants with PNAs of 0–7 and 7–28 days, respectively, at a fosfomycin MIC of 32 mg/L (Figure 4c). For all regimens, gestational ages, and PNAs, the bacterial stasis target (AUC:MIC >23)^11^ was met at predicted rates of greater than or equal to 95% until a fosfomycin MIC of 128 mg/L (Figure 4b,d).

**FIGURE 5:**
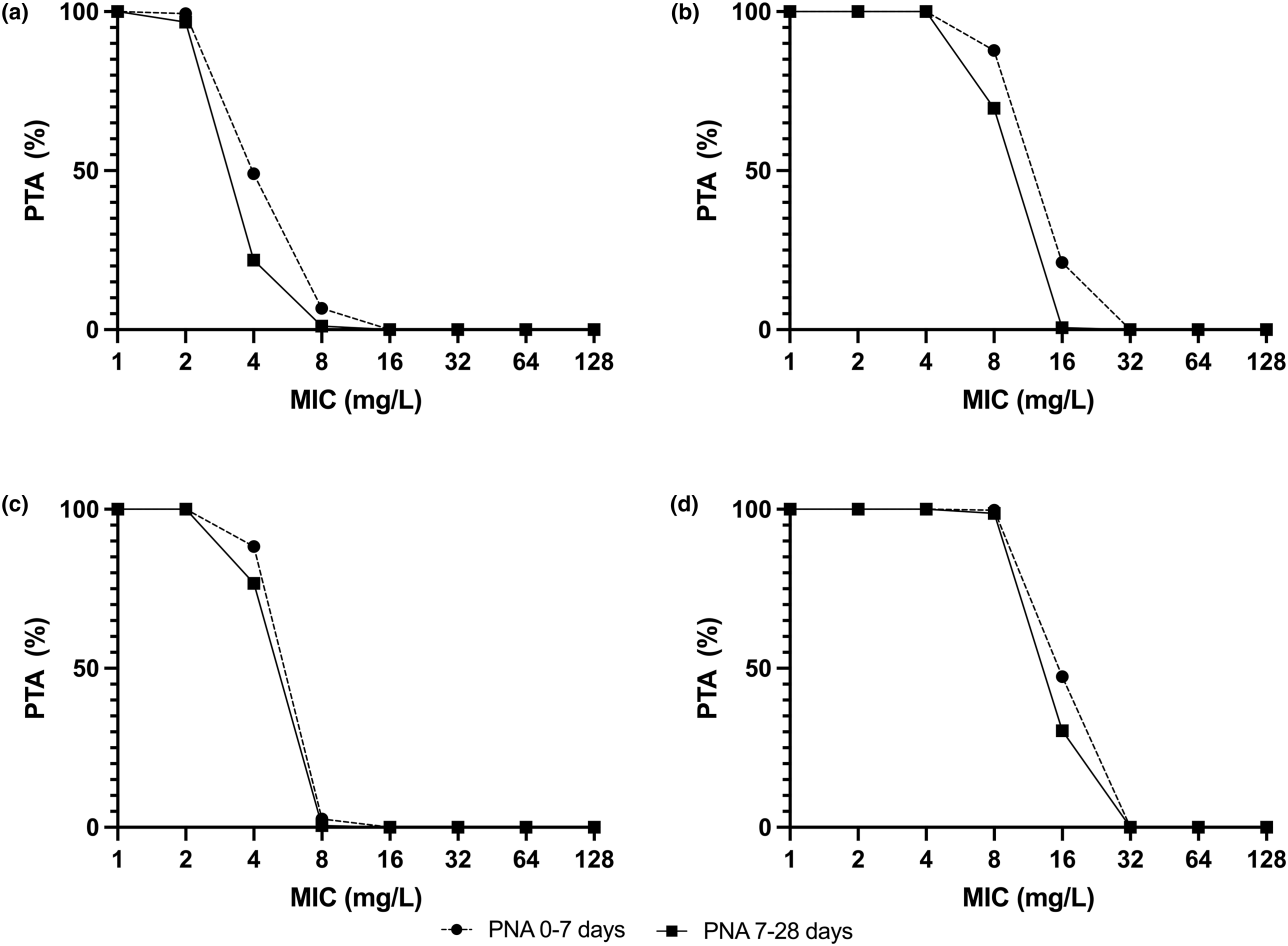
Probability of target attainment for amikacin monotherapy for amikacin 15 mg/kg IV q24h in neonates of PNA 0–7 days and 7–28 days. Panels (a and c) represent the target attainment for 1−log_10_ CFU/mL reduction (AUC:MIC >62.5). Panels (b and d) represent the target attainment for bacterial stasis (AUC:MIC >21.4). Panels (a and b) are determined in term neonates. Panels (c and d) are determined in neonates born at 30 weeks gestation. AUC, area under the curve; MIC, minimum inhibitory concentration; PNA, post‐natal age; PTA, probability of target attainment.

Amikacin target attainment was also determined using the clinical target of *C*_max_:MIC greater than 10.^12^ For all PNA and gestational groups, this target was met at greater than 99% attainment rate at an amikacin MIC of 16 mg/L, but 0% for an MIC of 32 mg/L. Using the plasma concentration at 5 min, replicating the likely captured *C*_max_ in a PK study, 100% target attainment was predicating for an amikacin MIC of 4 mg/L, but 0% for an MIC of 8 mg/L. For illustrative purposes, the difference between the PBPK‐predicted *C*_max_ and the concentration at 5 min is shown in Table S8. An alternative PTA analysis was conducted using the alternate AUC:MIC PK/PD targets used by EUCAST/USCAST^13^ in Figure 5. There is differential attainment of the stasis target with greater than 95% attainment (for both PNA groups) at an amikacin MIC of 8 mg/L for pre‐term infants, but at 4 mg/L for term infants. Greater than 95% attainment of the 1−log_10_ CFU reduction target was met at an amikacin MIC of 2 mg/L for all neonates.

Target attainment was determined for the proposed combination breakpoint for fosfomycin/amikacin described in our earlier work.^6^ In all groups, greater than 95% probability of attainment was achieved for fosfomycin and amikacin MICs above the line MIC_fosfomycin_* MIC_amikacin_ = 128 (i.e., fosfomycin and amikacin MIC combinations of 16 mg/L and 8 mg/L, 2 mg/L and 64 mg/L, etc.) as it appears in Table 2. Premature neonates achieved greater than 95% probability of attainment for fosfomycin and amikacin MICs along the line MIC_fosfomycin_* MIC_amikacin_ = 128 (Table S9).

**TABLE 2:** Probability of target attainment for the fosfomycin/amikacin combination breakpoint described in Darlow et al.^6^ across a range of fosfomycin and amikacin MICs for each of the PBPK model simulations in term neonates.

Amikacin MIC	Fosfomycin MIC							
	1	2	4	8	16	32	64	128
(a)								
1	100%	100.00%	100.00%	100.00%	100.00%	100.00%	99.40%	87.40%
2	100%	100.00%	100.00%	100.00%	100.00%	99.40%	87.40%	51.30%
4	100%	100.00%	100.00%	100.00%	99.40%	87.40%	51.30%	19.70%
8	100%	100.00%	100.00%	99.40%	87.40%	51.30%	19.70%	6.90%
16	100%	100.00%	99.40%	87.40%	51.30%	19.70%	6.90%	1.90%
32	100%	99.40%	87.40%	51.30%	19.70%	6.90%	1.90%	0%
64	99.40%	87.40%	51.30%	19.70%	6.90%	1.90%	0%	0%
128	87.40%	51.30%	19.70%	6.90%	1.90%	0%	0%	0%
(b)								
1	100%	100%	100%	100%	100%	100%	97.30%	68.00%
2	100%	100%	100%	100%	100%	97.30%	68.00%	23.20%
4	100%	100%	100%	100%	97.30%	68.00%	23.20%	6.30%
8	100%	100%	100%	97.30%	68.00%	23.20%	6.30%	1.10%
16	100%	100%	97.30%	68.00%	23.20%	6.30%	1.10%	0.40%
32	100%	97.30%	68.00%	23.20%	6.30%	1.10%	0.40%	0%
64	97.30%	68.00%	23.20%	6.30%	1.10%	0.40%	0%	0%
128	68.00%	23.20%	6.30%	1.10%	0.40%	0%	0%	0%
(c)								
1	100%	100%	100%	100%	100%	100%	100%	89.80%
2	100%	100%	100%	100%	100%	100%	89.80%	48.30%
4	100%	100%	100%	100%	100%	89.80%	48.30%	13.80%
8	100%	100%	100%	100%	89.80%	48.30%	13.80%	3.10%
16	100%	100%	100%	89.80%	48.30%	13.80%	3.10%	0.80%
32	100%	100%	89.80%	48.30%	13.80%	3.10%	0.80%	0%
64	100%	89.80%	48.30%	13.80%	3.10%	0.80%	0%	0%
128	89.80%	48.30%	13.80%	3.10%	0.80%	0%	0%	0%

*Note*: (a) Fosfomycin 100 mg/kg q12h and amikacin 15 mg/kg q24h in term neonates with PNA of 0–7 days; (b) Fosfomycin 100 mg/kg q12h and amikacin 15 mg/kg q24h in term neonates with PNA of 7–28 days; (c) Fosfomycin 150 mg/kg q12h and amikacin 15 mg/kg q24h in term neonates with PNA of 7–28 days.

Abbreviations: MIC, minimum inhibitory concentration; PBPK, physiologically‐based pharmacokinetic; PNA, post‐natal age.

## DISCUSSION

To our knowledge, this is the first PBPK model for fosfomycin and amikacin in infants, with high performance in adult, pediatric, and neonatal populations for both agents. Both models straightforwardly scaled from adults to pediatric populations when applying Simcyp's physiological and ontogenetic scaling relationships. However, both models required modification for scaling to neonates, as neither captured the observed change in *V*_d_. Although this was a relatively minor model adjustment for fosfomycin, amikacin required a more significant model modification to capture the change in neonatal *V*_d_ seen from observed data. This change in amikacin *V*_d_ as neonates age is well‐documented,^45^ but the mechanism is not understood. Further characterization of this mechanism is required in order to incorporate this more generally in pediatric PBPK models. In the absence of an understood physiological mechanism, we adjusted the tissue partitioning scaling constant in neonates to produce a well‐functioning neonatal model.

Fosfomycin and amikacin, in combination, is currently being testing in an international multicenter trial,^46^ with regimens of amikacin (15 mg/kg i.v. bolus q24h) and fosfomycin (100 mg/kg i.v. bolus q12h for neonates with PNA of 0–7 days, increasing to 150 mg/kg for those with PNA of 7–28) as simulated by ourselves. The PTA analyses we performed, therefore, are re‐assuring for both of these regimens. Fosfomycin achieved a high probability of target attainment at the adult EUCAST fosfomycin monotherapy breakpoint.^47^ Although our PTA analysis did not predict a categorical change in target attainment for the higher fosfomycin regimen in neonates aged 7–28 days, it did markedly increase the probability of target attainment at a fosfomycin MIC of 32 mg/L to 85.1% from 30.7%, justifying this recommendation of an increased dose for the older age group. Amikacin breakpoints have a degree of uncertainty, with recent revisions to the breakpoints and variation between breakpoint setting bodies.^13^, ^47^ However, the amikacin PTAs predicted here with AUC:MIC target were comparable to or in excess of those predicted for adults.^13^

The regimens met our proposed combination breakpoints at lower MIC combinations, but it is worth noting that this threshold is more conservative, derived from the ability of the regimen to affect a sustained 5‐log_10_ kill without emergence of resistance in the hollow‐fiber infection model,^6^ which is a higher PD target than those calculated for fosfomycin and amikacin monotherapy. Given the risk of emergence of resistance with fosfomycin monotherapy,^48^ along with an apparently different (but as yet unquantified) PK/PD target for suppression of emergence of resistance,^49^ means that such higher PD targets need to be considered for sustainable use of fosfomycin.

There are some limitations to the conclusions from the simulations. In particular, there are limited PK data available from pre‐term neonates for both drugs. Although extrapolation beyond the observed data is possible in PBPK modeling, the conclusions for the simulations in pre‐term infants should be interpreted with caution, given the potential uncertainty in this extrapolation.

A limitation of traditional breakpoint determination is that they are based on the possible adult PK profiles characterized by PopPK models. The data that inform these PopPK models are typically from healthy volunteers or specific patient groups. Therefore, the attainable PKs and the PTA that led to the definition of breakpoints are, strictly speaking, relevant for these populations only. Yet in clinical practice, these breakpoints are relied upon in special populations that do not have the same physiology, including pediatric and neonatal populations.

This is a recognized limitation by leading breakpoint setting committees (e.g., EUCAST and CLSI), but determining new breakpoints for each population for each drug using the PopPK method would require extensive PK trials determining to create PopPK models for each special population. Here, we propose an alternative model. Pediatric PBPK modeling is finding increasing application in drug development and dose selection^50^ and this study illustrates how validated pediatric PBPK models can simulate drug exposures in these special populations and determine predicted PTAs, in the absence of specific PK data in these populations. We have demonstrated this for neonatal patients with the two models described here, but they could be applied to other patient groups, dependent on validation of interindividual variability in a given population.

The utility of combination agents for therapeutic benefit is currently a contentious issue, largely due lack of positive clinical data to support use and inconsistency in framing the potential benefit. One such framing of the potential benefit is the extension of efficacy to pathogens normally nonsusceptible to both agents, as demonstrated with previous work by ourselves.^6^ Current conceptualization of antimicrobial susceptibility is based around single dimension breakpoints for individual agents, but more complex multidimensional susceptibility breakpoints may be the reality, such as the one described in our previous work.^6^ However these combination breakpoints are defined, parallel PBPK models are a useful method to simultaneously predict drug exposures of two or more antimicrobials in a simulated patient cohort in a way that is difficult for PopPK models constructed with different variables and underlying covariance matrices.

The PBPK model and simulations we have demonstrated here, therefore, represents a potential template for predicting PTA for combination antimicrobials as well as assessing the PTA for traditional monotherapy PK/PD targets. In the specific context of fosfomycin and amikacin in neonates, it can give confidence to the selected regimens of fosfomycin and amikacin for use in the treatment of neonatal sepsis. However, these principles can be applied more broadly in the assessment of appropriate drug exposures of antimicrobials (alone or in combination) in special populations to ensure efficacious and sustainable use of these drugs in these populations.

## AUTHOR CONTRIBUTIONS

All authors wrote the manuscript. C.A.D., N.P., R.W.P., and W.H. designed the research. C.A.D. performed the research. C.A.D. and N.P. analyzed the data.

## FUNDING INFORMATION

C.D. was funded to conduct this work by the National Institute for Healthcare Research (Award Ref. CL‐2022‐07‐001) and the North West England Medical Research Council Fellowship Scheme in Clinical Pharmacology and Therapeutics, which is funded by the Medical Research Council (Award Ref. MR/N025989/1), Roche Pharma, Eli Lilly and Company Limited, UCB Pharma, Novartis, the University of Liverpool, and the University of Manchester.

## CONFLICT OF INTEREST STATEMENT

C.D. and W.H. have received funding for other projects from the Global Antibiotic Research and Development Partnership (GARDP). N.P. and R.P. were employees of Roche for at least part of the duration of this work. W.H. holds or has recently held research grants with UKRI, EU (FP7, IMI‐1, IMI‐2), Wellcome, F2G, Spero Therapeutics, Antabio, Pfizer, Allecra, Bugworks, Phico Therapeutics, BioVersys, Global Antimicrobial Research and Development Partnership (GARDP), and NAEJA‐RGM. He is (or has recently been) a consultant for Appili Therapeutics, F2G, Spero Therapeutics, Pfizer, GSK, Phico Therapeutics, Pulmocide, and Mundipharma Research Ltd. He was a member of the Specialist Advisory Committee for GARDP (2020–2023), a member of the British Society for Antimicrobial Chemotherapy (BSAC) Breakpoint Committee (2020–2023), a member of Health Technology Appraisal (HTA) Prioritization Committee for hospital care, and is the Specialty National Co‐lead for Infection for the National Institute of Health Research (NIHR) (2020‐current). All authors have no further conflicts of interests to declare.

## Supporting information


Tables S1–S9.
