# Supplementary material for: Development and application of neonatal physiology‐based pharmacokinetic models of amikacin and fosfomycin to assess pharmacodynamic target attainment
Source: CPT Pharmacometrics Syst Pharmacol. 2024 Jan 3;13(3):464–75. doi: 10.1002/psp4.13097 (PMC10941605; doi:10.1002/psp4.13097)
Supplement: Supplementary file 1 — Tables S1–S9. [file PSP4-13-464-s001.docx]

**Development and application of neonatal physiology-based pharmacokinetic models of amikacin and fosfomycin to assess pharmacodynamic target attainment - Supplementary Data**

**Supplementary Tables**

| Source Data set | Simulated regimen | Observed AUC (mg/L*h) | Predicted AUC (mg/L*h) | Observed Cmax (mg/L) | Predicted Cmax (mg/L) | Observed: predicted AUC ratio | Observed: predicted Cmax ratio |
| --- | --- | --- | --- | --- | --- | --- | --- |
| Segre et al^1^ (n=5) | 50mg/kg IV bolus | 456.39 | 428.91 | 349.16 | 371 | 1.06 | 0.94 |
| Bergan et al^2^ (n=12) | 3g IV bolus | 414.33 | 363.48 | 370.6 | 313 | 1.14 | 1.18 |
| Cadórniga et al^3^ (n=6) | 500mg IV bolus | 53.11 | 60.58 | 29.2 | 27 | 0.88 | 1.08 |
| Kwan et al^4^ (n=1) | 500mg IV bolus q6h (first dose interval) | 55.44 | 56.63 | 28 | 28.5 | 0.98 | 0.98 |
| Kwan et al^4^ (n=1) | 500mg IV bolus q6h (7th dose interval) | 69.68 | 61.04 | 36 | 42.3 | 1.14 | 0.85 |
| Kirby et al^5^ (n=4) | 2g IV 15min infusion | 145 | 244.66 | 87.98 | 163.22 | 0.59 | 0.54 |
| Kirby et al^5^ (n=4) | 1g IV 15min infusion | 90.78 | 122.39 | 46.07 | 81.64 | 0.74 | 0.56 |
| Kirby et al^5^ (n=4) | 500mg IV 15min infusion | 52.02 | 61.26 | 26.33 | 40.84 | 0.85 | 0.64 |
| Kirby et al^5^ (n=4) | 250mg IV 15min infusion | 36.2 | 30.69 | 14.31 | 20.45 | 1.18 | 0.70 |
| Kirby et al^5^ (n=2) | 4g IV 30min infusion q6h | 962.7 | 943.42 | 194.39 | 267.91 | 1.02 | 0.73 |
| Kirby et al^5^ (n=4) | 500mg IV bolus q6h (first dose interval) | 51.2 | 57.16 | 28.1 | 40.84 | 0.90 | 0.69 |
| Kirby et al^5^ (n=4) | 500mg IV bolus q6h (sixth dose interval)) | 66.9 | 61.61 | 36 | 42.61 | 1.09 | 0.84 |
| Goto et al^6^ (n=7)* | 20mg/kg IV bolus | 154.3 | 170.79 | 130.5 | 137 | 0.90 | 0.95 |
| Goto et al^6^ (n=7)* | 40mg/kg IV bolus | 271.4 | 341.58 | 260.37 | 274 | 0.79 | 0.95 |
| Lastra et al^7^ (n=9) | 30mg/kg IV bolus | 217.1 | 250.04 | 163.2 | 131 | 0.87 | 1.25 |
| Wenzler et al ^8^(n=27) | 1g IV 1h infusion | 117.1 | 122.25 | 43.51 | 40.8 | 0.96 | 1.07 |
| Wenzler et al ^8^(n=27) | 8g IV 1h infusion | 1068 | 985.72 | 365.97 | 344 | 1.08 | 1.06 |
|  |  |  | Weighted geometric mean ratio | | | 0.96 | 0.97 |

Table S1 –Observed fosfomycin PK data in adults compared to the mean PK predicted by the adult fosfomycin PBPK model. * indicates PBPK simulations in the Simcyp Japanese Adult population. All other simulations used the Simcyp Caucasian Adult population. The summary geometric mean was weighted by the number of participants in each dataset.

| Source Data set | Simulated regimen | Age range (years) | Observed AUC (mg/L*h) | Predicted AUC (mg/L*h) | Observed Cmax (mg/L) | Predicted Cmax (mg/L) | Observed: predicted AUC ratio | Observed: predicted Cmax ratio |
| --- | --- | --- | --- | --- | --- | --- | --- | --- |
| Guggenbichler et al^9^ (n=10) | 25mg/kg IV bolus | 3 - 8 | 123.9 | 120.69 | 102 | 86.5 | 1.03 | 1.18 |
| Guggenbichler et al^9^ (n=10) | 50mg/kg IV bolus | 3 - 8 | 288.1 | 248.36 | 194 | 173 | 1.16 | 1.12 |
| Iwai et al^10^ (n=4)* | 25mg/kg IV bolus | 3 - 8 | 82 | 97.29 | 55.3 | 69.8 | 0.84 | 0.79 |
| Iwai et al^10^ (n=4)* | 50mg/kg IV bolus | 4 - 10 | 176 | 213.14 | 118.8 | 146 | 0.83 | 0.81 |
| Iwai et al^10^ (n=5)* | 25mg/kg IV 1h infusion | 3 - 7 | 114.8 | 139.18 | 58.1 | 74.30 | 0.82 | 0.83 |
| Iwai et al^10^ (n=2)* | 50mg/kg IV 1h infusion | 8 - 9 | 276.9 | 304.03 | 150.3 | 157.02 | 0.91 | 1.02 |
|  |  |  |  | Weighted geometric mean | | | 0.98 | 0.99 |

Table S2 – Comparison of observed fosfomycin PK data in paediatric populations from datasets compared to the mean PK profile predicted by the paediatric fosfomycin PBPK model. * indicates PBPK simulations in the Simcyp Japanese paediatric population. All other simulations were in the Simcyp Caucasian paediatric population. The summary geometric mean was weighted by the number of participants in each dataset.

| Source Data set | Simulated regimen | Age range (days) | Observed AUC (mg/L*h) | Predicted AUC (mg/L*h) | Observed Cmax (mg/L) | Predicted Cmax (mg/L) | Observed: predicted AUC ratio | Observed: predicted Cmax ratio |
| --- | --- | --- | --- | --- | --- | --- | --- | --- |
| Guggenbichler et al^9^ (n=5) | 25mg/kg IV bolus | Not available* | 204.1 | 247.29 | 62 | 72.8 | 0.83 | 0.85 |
| Kane et al^11^ (n=61) | 100mg/kg IV bolus | 0 – 23 | 1238 | 1172.97 | 317.55 | 346 | 1.06 | 0.92 |
| Molina et al^12^ (n=6) ‡ | 50mg/kg IV bolus | 1 – 3 | 719.6 | 774.05 | 98.58 | 204 | 0.93 | 0.48 |
| Molina et al^12^ (n=5) ‡ | 50mg/kg IV bolus | 20 – 34 | 531.2 | 730.01 | 96.5 | 202 | 0.73 | 0.48 |
| Guibert et al^13^ (n=5)† | 100mg/kg IV 30min infusion | 16 - 30 | 1034.4 | 1332.02 | 136.4 | 363.04 | 0.78 | 0.38 |
| Guibert et al^13^ (n=5)† | 100mg/kg IV 2h infusion | 7 - 53 | 1194.6 | 1330.77 | 133 | 252.63 | 0.90 | 0.53 |
|  |  |  |  | Weighted geometric mean | | | 0.95 | 0.71 |

Table S3 – Comparison of observed fosfomycin PK data in neonatal populations from datasets compared to the mean PK profile predicted by the original neonatal fosfomycin PBPK model (Kp scalar = 1). * Age range of participants not available in source paper. Comparator PBPK simulation used 0 – 3 days. The summary geometric mean was weighted by the number of participants in each dataset. ‡ The Molina et al dataset included a large number of moderately premature (mean 36 and 34 weeks gestational age, respectively) low birth weight (all <2kg) individuals. Both datasets were simulated with the Pre-term Simcyp population at their reported gestational age, but the simulated populations did not capture the body weight demographics † The Guibert et al data set did not provide individual values, but provided summary AUC and Cmax for each population. The observed values here are these original values calculated by Guibert et al. As the exact observed time of Cmax was not specified, we compared predicted concentration at 5 min after the end of the infusion

| Source Data set | Simulated regimen | Age range (days) | Observed AUC (mg/L*h) | Predicted AUC (mg/L*h) | Observed Cmax (mg/L) | Predicted Cmax (mg/L) | Observed: predicted AUC ratio | Observed: predicted Cmax ratio |
| --- | --- | --- | --- | --- | --- | --- | --- | --- |
| Guggenbichler et al^9^ (n=5) | 25mg/kg IV bolus | Not available* | 204.1 | 297.25 | 62 | 72.1 | 0.69 | 0.86 |
| Kane et al^11^ (n=61) | 100mg/kg IV bolus | 0 – 23 | 1238 | 1088.13 | 317.55 | 315 | 1.14 | 1.01 |
| Molina et al^12^ (n=6) ‡ | 50mg/kg IV bolus | 1 – 3 | 719.6 | 765.19 | 98.58 | 186 | 0.94 | 0.53 |
| Molina et al^12^ (n=5) ‡ | 50mg/kg IV bolus | 20 – 34 | 531.2 | 663.46 | 96.5 | 183 | 0.80 | 0.53 |
| Guibert et al^13^ (n=5)† | 100mg/kg IV 30min infusion | 16 - 30 | 1034.4 | 1334 | 136.4 | 284 | 0.78 | 0.48 |
| Guibert et al^13^ (n=5)† | 100mg/kg IV 2h infusion | 7 - 53 | 1194.6 | 1333.22 | 133 | 212 | 0.90 | 0.63 |
|  |  |  |  | Weighted geometric mean | | | 1.03 | 0.86 |
|  |  |  |  | Modified weighted geometric mean 1 | | | 0.98 | 0.79 |
|  |  |  |  | Modified weighted geometric mean 2 | | | 1.06 | 0.92 |
|  |  |  |  | Modified weighted geometric mean 3 | | | 1.01 | 0.87 |

Table S4 – Comparison of observed fosfomycin PK data in neonatal populations from datasets compared to the mean PK profile predicted by the adjusted neonatal fosfomycin PBPK model (Kp scaler = 1.2). * Age range of participants not available in source paper. Comparator PBPK simulation used 0 – 3 days. The summary geometric mean was weighted by the number of participants in each dataset. ‡ The Molina et al dataset included a large number of moderately premature (mean 36 and 34 weeks gestational age, respectively) low birth weight (all <2kg) individuals. Both datasets were simulated with the Pre-term Simcyp population at their reported gestational age, but the simulated populations did not capture the body weight demographics † The Guibert et al data set did not provide individual values, but provided summary AUC and Cmax for each population. The observed values here are these original values calculated by Guibert et al. As the exact observed time of Cmax was not specified, we compared at 5 min after the end of the infusion. For illustrative purposes, modified weighted geometrics means are given for calculations where (1) Kane et al is given 50% weighting; (2) Molina et al is excluded; (3) both.

| Source Data set | Simulated regimen | Observed AUC (mg/L*h) | Predicted AUC (mg/L*h) | Observed Cmax (mg/L) | Predicted Cmax (mg/L) | Observed: predicted AUC ratio | Observed: predicted Cmax ratio |
| --- | --- | --- | --- | --- | --- | --- | --- |
| Lode et al^14^ (n=12) | 7.5mg/kg 1h IV infusion | 95.13 | 71.11 | 37.5 | 30 | 1.34 | 1.25 |
| Adamis et al^15^ (n=3) | 500mg IV bolus | 45.88 | 65.44 | 33.3 | 47.5 | 0.70 | 0.70 |
| Garraffo et al^16^ (n=6) | 7.5mg/kg 30min IV infusion | 76.18 | 72.89 | 35 | 39.49 | 1.05 | 0.89 |
| Garraffo et al^16^ (n=6) | 15mg/kg 30min IV infusion | 164.8 | 145.78 | 77 | 78.98 | 1.13 | 0.97 |
| Wise et al^17^ (n=10) | 500mg IV bolus | 77.72 | 54.99 | 41.62 | 32.5 | 1.41 | 1.28 |
| Clarke et al^18^ (n=5) | 3.33mg/kg 60min IV infusion then 3mg/kg 180min IV infusion | 72.69 | 59.49 | 15.05 | 13.4 | 1.22 | 1.12 |
|  |  |  | Weighted geometric mean | | | 1.21 | 1.09 |

Table S5 – Comparison of observed amikacin PK data in healthy volunteer adults from datasets compared to the mean PK profile predicted by the adult amikacin PBPK model. The summary geometric mean was weighted by the number of participants in each dataset.

| Source Data set | Simulated regimen | Age range (years) | Observed AUC (mg/L*h) | Predicted AUC (mg/L*h) | Observed Cmax (mg/L) | Predicted Cmax (mg/L) | Observed: predicted AUC ratio | Observed: predicted Cmax ratio |
| --- | --- | --- | --- | --- | --- | --- | --- | --- |
| Cleary et al^19^ (n=12) | 5mg/kg 30min IV infusion | 1 – 16 | 37.53 | 35.28 | 29.3 | 22.4 | 1.06 | 1.31 |
| Cleary et al^19^ (n=12) | 5mg/kg 60min IV infusion | 1 – 16 | 42.83 | 35.25 | 18.1 | 17.7 | 1.22 | 1.02 |
| Cleary et al^19^ (n=12) | 5mg/kg 60min IV infusion q8h | 1 – 16 | 36.78 | 34.28 | 17.2 | 17.6 | 1.07 | 0.98 |
| Kafetzis et al^20^ (n=13) | 20mg/kg 30min IV infusion | 1 – 14 | 90.3 | 59.21 | 36.5 | 35.8 | 1.53 | 1.02 |
| Motohiro et al^21^ (n=3)* | 2mg/kg 30min IV infusion | 7 – 11 | 14.58 | 12.75 | 9.23 | 8.12 | 1.14 | 1.14 |
| Motohiro et al^21^ (n=3)* | 4mg/kg 60min IV infusion | 7 – 11 | 30.51 | 25.48 | 13.67 | 12.9 | 1.20 | 1.06 |
| Nanri et al^22^ (n=4)* | 2.5mg/kg 30min IV infusion | 4 – 6 | 11.64 | 15.25 | 9.68 | 10.0 | 0.76 | 0.97 |
| Nanri et al^22^ (n=3)* | 2.5mg/kg 60min IV infusion | 4 – 11 | 14.81 | 15.59 | 9.425 | 7.98 | 0.95 | 1.18 |
| Nanri et al^22^ (n=2)* | 2.5mg/kg 60min IV infusion | <1 | 20.96 | 18.16 | 10.75 | 8.34 | 0.59 | 1.29 |
|  |  |  |  | Weighted geometric mean | | | 1.13 | 1.08 |

Table S6 – Comparison of observed amikacin PK data in paediatric populations from datasets compared to the mean PK profile predicted by the paediatric amikacin PBPK model. * indicates PBPK simulations in the Simcyp Japanese paediatric population. All other simulations were in the Simcyp Caucasian paediatric population. The summary geometric mean was weighted by the number of participants in each dataset.

| Source Data set | Simulated regimen | Age range (days) | Observed AUC (mg/L*h) | Predicted AUC (mg/L*h) | Observed Cmax (mg/L) | Predicted Cmax (mg/L) | Observed: predicted AUC ratio | Observed: predicted Cmax ratio |
| --- | --- | --- | --- | --- | --- | --- | --- | --- |
| Hashira et al^23^ (n=2) | 5mg/kg 60min IV infusion | 1 - 5 | 106.3 | 60.79 | 14.05 | 14.64 | 1.75 | 0.96 |
| Hashira et al^23^ (n=1)* | 6mg/kg 60min IV infusion | 7 | 134.3 | 123.84 | 26.1 | 17.56 | 1.08 | 1.49 |
| Hashira et al^23^ (n=2) | 7.5mg/kg 60min IV infusion | 0 - 5 | 184.25 | 119.02 | 32.7 | 21.95 | 1.55 | 1.49 |
| Iwai et al^24^ (n=1)* | 3mg/kg 30min IV IV infusion | 10 | 27.14 | 42.23 | 7.4 | 9.15 | 0.64 | 0.81 |
| Iwai et al^24^ (n=2) | 3mg/kg 30min IV IV infusion | 9 - 23 | 24.08 | 32.05 | 7.85 | 10.25 | 0.75 | 0.77 |
| Iwai et al^24^ (n=4) | 6mg/kg 30min IV infusion | 10 - 25 | 57.71 | 63.80 | 20.88 | 20.48 | 0.90 | 1.02 |
| Iwai et al^24^ (n=1) | 6mg/kg 60min IV infusion | 20 | 83.8 | 60.26 | 19.4 | 16.66 | 1.39 | 1.16 |
| Motohiro et al^21^ (n=3) | 3mg/kg 30min IV infusion | 2 - 27 | 28.85 | 28.00 | 7.61 | 10.30 | 1.03 | 0.74 |
| Motohiro et al^21^ (n=1) | 6mg/kg 30min IV infusion | 27 | 33.83 | 50.42 | 14.1 | 20.10 | 0.67 | 0.70 |
| Motohiro et al^21^ (n=2) | 6mg/kg 60min IV infusion | 27 - 28 | 44.85 | 50.86 | 17.6 | 16.55 | 0.88 | 1.06 |
| Nanri et al^22^ (n=1) | 3mg/kg 30min IV infusion | 0 | 35.55 | 31.16 | 8.4 | 10.52 | 1.14 | 0.80 |
| Nanri et al^22^ (n=2) | 3mg/kg 60min IV infusion | 0 | 29.7 | 30.27 | 6.85 | 8.76 | 0.98 | 0.78 |
| Nanri et al^22^ (n=3) | 6mg/kg 30min IV infusion | 0 - 11 | 75.05 | 60.27 | 24.2 | 21.03 | 1.25 | 1.15 |
| Nanri et al^22^ (n=1) | 6mg/kg 60min IV infusion | 0 | 37.7 | 44.40 | 15.5 | 17.52 | 0.85 | 0.88 |
| Nishimura et al^25^ (n=4) | 3mg/kg 30min IV infusion | 2 - 8 | 27.24 | 32.04 | 9.03 | 10.54 | 0.85 | 0.86 |
| Nishimura et al^25^ (n=3) | 6mg/kg 30min IV infusion | 1 - 5 | 44.18 | 65.09 | 15.43 | 21.11 | 0.68 | 0.73 |
|  |  |  |  | Weighted geometric mean | | | 0.98 | 0.93 |

Table S7 – Comparison of observed amikacin PK data in neonatal populations from datasets compared to the mean PK profile predicted by the neonatal amikacin PBPK model. All simulations were run using the Simcyp Japanese paediatric population *Indicates dataset from pre-term neonates – these were run using the Simcyp Pre-term population with gestational age of 25 weeks for Hishira et al and 32 weeks for Iwai et al. All other infants were term (36 weeks or greater). The summary geometric mean was weighted by the number of participants in each dataset.

| Simulated population | Mean C_max_ (mg/L) | Mean C_5min_ (mg/L) | Mean C_max_ - C_5min_ (mg/L) |
| --- | --- | --- | --- |
| Term (PNA 0-7d | 219.83 | 47.44 | 172.39 |
| Term (PNA 7-28d) | 213.87 | 48.17 | 165.70 |
| Pre-term (PNA 0-7d) | 237.95 | 53.24 | 184.71 |
| Pre-term (PNA 7-28d) | 238.68 | 52.61 | 186.06 |

**Table S8** – Mean maximum plasma concentration (C_max_), plasma concentration at 5 min (C_5min_), and calculated difference in PBPK model output of simulated administration of amikacin in four cohorts (n=1000 in each) of term and pre-term neonatal populations with post-natal age (PNA) of 0-7 days and 7-28 days.

| A. | Fosfomycin MIC | | | | | | | |
| --- | --- | --- | --- | --- | --- | --- | --- | --- |
| Amikacin MIC | 1 | 2 | 4 | 8 | 16 | 32 | 64 | 128 |
| 1 | 100% | 100% | 100% | 100% | 100% | 100% | 100% | 99.50% |
| 2 | 100% | 100% | 100% | 100% | 100% | 100% | 99.50% | 90.10% |
| 4 | 100% | 100% | 100% | 100% | 100% | 99.50% | 90.10% | 44.20% |
| 8 | 100% | 100% | 100% | 100% | 99.50% | 90.10% | 44.20% | 3.20% |
| 16 | 100% | 100% | 100% | 99.50% | 90.10% | 44.20% | 3.20% | 0% |
| 32 | 100% | 100% | 99.50% | 90.10% | 44.20% | 3.20% | 0% | 0% |
| 64 | 100% | 99.50% | 90.10% | 44.20% | 3.20% | 0% | 0% | 0% |
| 128 | 99.50% | 90.10% | 44.20% | 6.90% | 0% | 0% | 0% | 0% |

| B. | Fosfomycin MIC | | | | | | | |
| --- | --- | --- | --- | --- | --- | --- | --- | --- |
| Amikacin MIC | 1 | 2 | 4 | 8 | 16 | 32 | 64 | 128 |
| 1 | 100% | 100% | 100% | 100% | 100% | 100% | 100% | 98.50% |
| 2 | 100% | 100% | 100% | 100% | 100% | 100% | 98.50% | 78.30% |
| 4 | 100% | 100% | 100% | 100% | 100% | 98.50% | 78.30% | 27.60% |
| 8 | 100% | 100% | 100% | 100% | 98.50% | 78.30% | 27.60% | 0.60% |
| 16 | 100% | 100% | 100% | 98.50% | 78.30% | 27.60% | 0.60% | 0% |
| 32 | 100% | 100% | 98.50% | 78.30% | 27.60% | 0.60% | 0% | 0% |
| 64 | 100% | 98.50% | 78.30% | 27.60% | 0.60% | 0% | 0% | 0% |
| 128 | 98.50% | 78.30% | 27.60% | 0.60% | 0.40% | 0% | 0% | 0% |

| C. | Fosfomycin MIC | | | | | | | |
| --- | --- | --- | --- | --- | --- | --- | --- | --- |
| Amikacin MIC | 1 | 2 | 4 | 8 | 16 | 32 | 64 | 128 |
| 1 | 100% | 100% | 100% | 100% | 100% | 100% | 100% | 99.90% |
| 2 | 100% | 100% | 100% | 100% | 100% | 100% | 99.90% | 94.60% |
| 4 | 100% | 100% | 100% | 100% | 100% | 99.90% | 94.60% | 60.10% |
| 8 | 100% | 100% | 100% | 100% | 99.90% | 94.60% | 60.10% | 9.60% |
| 16 | 100% | 100% | 100% | 99.90% | 94.60% | 60.10% | 9.60% | 0% |
| 32 | 100% | 100% | 99.90% | 94.60% | 60.10% | 9.60% | 0% | 0% |
| 64 | 100% | 99.90% | 94.60% | 60.10% | 9.60% | 0% | 0% | 0% |
| 128 | 99.90% | 94.60% | 60.10% | 9.60% | 0% | 0% | 0% | 0% |

**Table S9 –** Probability of target attainment for the fosfomycin/amikacin combination breakpoint described in Darlow et al^6^ across a range of fosfomycin and amikacin MICs for each of the PBPK model simulations in pre-term neonates. A – Fosfomycin 100mg/kg q12h and amikacin 15mg/kg q24h in pre-term neonates with PNA of 0-7 days; B – Fosfomycin 100mg/kg q12h and amikacin 15mg/kg q24h in pre-term neonates with PNA of 7-28 days; C – Fosfomycin 150mg/kg q12h and amikacin 15mg/kg q24h in pre-term neonates with PNA of 7-28 days;

**References**

1. Segre, G., Bianchi, E., Cataldi, A. & Zannini, G. Pharmacokinetic profile of fosfomycin trometamol (Monuril). *Eur. Urol.* **13 Suppl 1**, 56–63 (1987).

2. Bergan, T. Degree of absorption, pharmacokinetics of fosfomycin trometamol and duration of urinary antibacterial activity. *Infection* **18 Suppl 2**, S65-9 (1990).

3. Cadórniga, R., Diaz Fierros, M. & Olay, T. Pharmacokinetic Study of Fosfomycin and its Bioavailability. *Chemotherapy* **23**, 159–174 (1977).

4. Kwan, K. C., Wadke, D. A. & Foltz, E. L. Pharmacokinetics of Phosphonomycin in Man I:Intravenous Administration. *J. Pharm. Sci.* **60**, 678–685 (1971).

5. Kirby, W. M. M. Pharmacokinetics of Fosfomycin. *Chemotherapy* **23**, 141–151 (1977).

6. Goto, M., Sugiyama, M., Nakajima, S. & Yamashina, H. Fosfomycin kinetics after intravenous and oral administration to human volunteers. *Antimicrob. Agents Chemother.* **20**, 393–397 (1981).

7. Fernandez Lastra, C. *et al.* The influence of uremia on the accessibility of phosphomycin into interstitial tissue fluid. *Eur. J. Clin. Pharmacol.* **25**, 333–338 (1983).

8. Wenzler, E., Ellis-Grosse, E. J. & Rodvold, K. A. Pharmacokinetics, Safety, and Tolerability of Single-Dose Intravenous (ZTI-01) and Oral Fosfomycin in Healthy Volunteers. *Antimicrob. Agents Chemother.* **61**, e00775-17 (2017).

9. Guggenbichler, J., Kienel, G. & Frisch, H. [Fosfomycin, a new antibiotic drug (author’s transl)]. *Padiatr. Padol.* **13**, 429–436 (1978).

10. Iwai, N., Nakamura, H., Miyazu, M. & Watanabe, Y. [A study of the absorption and excretion of fosfomycin sodium in children]. *Jpn. J. Antibiot.* **44**, 345–356 (1991).

11. Kane, Z. *et al.* IV and oral fosfomycin pharmacokinetics in neonates with suspected clinical sepsis. *J. Antimicrob. Chemother.* **76**, 1855–1864 (2021).

12. Molina, M. A., Olay, T. & Quero, J. Pharmacodynamic Data on Fosfomycin in Underweight Infants during the Neonatal Period. *Chemotherapy* **23**, 217–222 (1977).

13. Guibert, M., Magny, J. F., Poudenx, F., Lebrun, L. & Dehan, M. [Comparative pharmacokinetics of fosfomycin in the neonate: 2 modes of administration]. *Pathol. Biol. (Paris)* **35**, 750–752 (1987).

14. Lode, H., Grunert, K., Koeppe, P. & Langmaack, H. Pharmacokinetic and Clinical Studies with Amikacin, a New Aminoglycoside Antibiotic. *J. Infect. Dis.* **134**, S316–S322 (1976).

15. Adamis, G. *et al.* Pharmacokinetic interactions of ceftazidime, imipenem and aztreonam with amikacin in healthy volunteers. *Int. J. Antimicrob. Agents* **23**, 144–149 (2004).

16. Garraffo, R., Drugeon, H. B., Dellamonica, P., Bernard, E. & Lapalus, P. Determination of optimal dosage regimen for amikacin in healthy volunteers by study of pharmacokinetics and bactericidal activity. *Antimicrob. Agents Chemother.* **34**, 614–621 (1990).

17. Wise, R., Walker, J. M. & Mitchard, M. A comparison of the pharmacokinetics of amikacin and gentamicin. *J. Antimicrob. Chemother.* **8 Suppl A**, 45–49 (1981).

18. Clarke, J. T., Libke, R. D., Regamey, C. & Kirby, W. M. M. Comparative pharmacokinetics of amikacin and kanamycin. *Clin. Pharmacol. Ther.* **15**, 610–616 (1974).

19. Cleary, T. G. *et al.* Amikacin pharmacokinetics in pediatric patients with malignancy. *Antimicrob. Agents Chemother.* **16**, 832 (1979).

20. Kafetzis, D. A. *et al.* Clinical and pharmacokinetic study of a single daily dose of amikacin in paediatric patients with severe Gram-negative infections. *J. Antimicrob. Chemother.* **27**, 105–112 (1991).

21. Motohiro, T. *et al.* [Pharmacokinetics of amikacin in children and neonates]. *Jpn. J. Antibiot.* **40**, 1200–14 (1987).

22. Nanri, S. *et al.* [A pharmacokinetic study in (mature and premature) neonates treated with amikacin through intravenous drip infusion]. *Jpn. J. Antibiot.* **40**, 1135–45 (1987).

23. Hashira, S., Koike, Y. & Fujii, R. [Fundamental study of amikacin in the newborn]. *Jpn. J. Antibiot.* **40**, 1129–34 (1987).

24. Iwai, N. *et al.* [Pharmacokinetics in neonates and infants following administration of amikacin]. *Jpn. J. Antibiot.* **40**, 1157–75 (1987).

25. Nishimura, T., Tabuki, K. & Takashima, T. [Pharmacokinetic and clinical studies on amikacin in neonates]. *Jpn. J. Antibiot.* **40**, 1183–91 (1987).
